# Ontogenetic variability in old and new collections of *Dicranophyllum gallicum* Grand’Eury from the late Palaeozoic of Europe

**DOI:** 10.3897/phytokeys.88.14042

**Published:** 2017-10-12

**Authors:** Jorik Van Der Pas, Linda Poppe, Isabel M. Van Waveren

**Affiliations:** 1 Naturalis Biodiversity Center, Postbus 9517, 2300 RA, Leiden, the Netherlands

**Keywords:** Early conifers, Carboniferous, Permian

## Abstract

*Dicranophyllum gallicum* Grand’Eury is described by means of a morphometric analysis of eighty two samples from various old and new localities in western and central Europe. Stem, leaf cushions, leaf scars, leaves, axillary structures and potential seeds are described in detail, and discussed in comparison to earlier studies. The encountered variability in size and structure is shown to be higher than what was described earlier. The organisation of the leaf cushion and scar density vary gradually with the stem width, while stratigraphic position and ecology do not relate to it. It is concluded that the described variability represents an ontogenetic feature rather than a phylogenetic or ecologic one. The juvenile plants are characterised by small stems, a high leaf scar density and elongated leaf cushions with a dominant apical field, while mature specimens are characterized by a wide stem, a relatively low leaf scar density and relatively wide leaf cushions with a pronounced basal field. Axillary shoots and potential seeds of *D. gallicum* are described and illustrated in detail for the first time. A reconstruction based on the studied material is presented.

## Introduction

*Dicranophyllum gallicum* Grand’Eury (1877) is a characteristic species of the late Mississippian to early Permian of Euramerica (Wagner 2005). The genus is typified by bifurcating leaves, inserted on the stem in helical arrangement, forming rhomboidal leaf cushions (Grand’Eury 1877). It has characters reminiscent of several higher taxonomic groups. The needle-like leaves may be associated to conifers, their bifurcations are reminiscent of ginkgophytes and the leaf cushions are found in lycophytes. *Dicranophyllum gallicum* Grand’Eury, the type species of the genus, is characterized by short, tough leaves bifurcating twice, with a central vein and two lateral stomatiferous furrows (Barthel 1977, Meyen and Smoller 1986). *Dicranophyllum gallicum* variatio *parchemineyi* was described by Renault and Zeiller (1888) to have a double row of small conical seeds attached to the unbifurcated base of the leaf (Renault and Zeiller 1888).

Given the divergent set of properties and the paucity of fertile specimens, taxonomic placement is difficult. Indeed, the genus is presented as a ginkgophyte in Taylor et al. (2009), while Anderson et al. (2007), following Cleal (1993), presented it as a member of the within the conifers. In the past, Grand’Eury (1877) compared *Dicranophyllum* to Salisburya (Ginkgo), but considered it as representing a new Paleozoic group of conifers. In the absence of insight in the internal organisation of the seeds, positioning within the cordaites, the cycads or the conifers was considered impossible to establish, but the conifers were preferred because of the position of the seeds (Renault and Zeiller 1888). Neuburg (1948) placed the species within the Ginkgoales. Němejc (1959) put *Dicranophyllum* and *Trichopitys* together in the new class , considering them to be intermediate between ginkgophytes and conifers, while having descended from the pteridosperms. Meyen and Smoller (1986) partly adopted this suggestion by assigning *Dicranophyllum* and *Trichopitys* to the order of the , which was placed within the Coniferopsida. Meyen (1987) stressed that the microdenticulate leaf margin observed in *D. gallicum* was absent in Ginkgoopsida, which pointed towards an affiliation with the Coniferopsida and the . Archangelsky and Cúneo (1990) described *Polyspermophyllum sergii*, which shows similarities to both *Trichopitys* and *Dicranophyllum*. They created two separate families and within the . Considering it difficult to relate the to either the Cycadopsida or the Coniferopsida, they interpreted them as an order of primitive gymnosperms. A phylogenetic analysis has indicated that *Dicranophyllum hallei* is represented by a branch basal to all earliest conifers, the Voltziales (Rothwell et al. 2005)

The present contribution provides a detailed description of *Dicranophyllum gallicum* specimens from French, German and new Czech collections. Potential fertile organs are documented in detail for the first time and strong variability within the species is shown to relate to the plants ontogeny rather than its phylogeny or ecology.

## Materials and methods

A total of 82 samples holding *Dicranophyllum gallicum* are described in this study (Suppl. material 1). These samples were assembled from the collections of the Museum d'Histoire Naturelle Jacques de la Comble in Autun, the Museum d'Histoire Naturelle in Paris, the collection at the Naturhistorisches Museum Schloss Bertholdsburg, Schleusingen, the collections at the Naturkundemuseum in Berlin, the Paläontologi Museum in Munich, the collections at the National Museum of Natural History in Prague (where the Lubná samples came from a new collection), the Czech Geological Survey in Prague and the Faculty of Geosciences of the Utrecht University in the Netherlands. The samples came from Ronchamp, Mont Pelé, Saint Etienne, Commentry and Lodève in France, Kladno, Knoviz and the new locality Lubná in Czeck Republic, and Zwickau, Sperbersbach, Kammerberg, Oelsnitz, Oberhof, Winnweiler and Rotterode in Germany (Table 1).

**Table 1.** Stratigraphy, age and lithology of the different localities.

**Locality**	**Country**	**Stratigraphic unit**	**Lithology**	**Age**
Kladno	Czech Republic	Radnice Member	tuff	Westphalian C (Roscher and Schneider 2006)
Lubná, Filip II quarry	Czech Republic	Radnice Member	tuff	Westphalian C (Roscher and Schneider 2006)
Zwickau	Germany	Zwickau Formation	volcanics	Westphalian D-Cantabrian (Schneider and Romer 2010)
Ronchamp	France	?	coaly mudstone, black coal	Stephanian (Aubouin 1965)
Mont-Pelé	France	? Mont-Pelé Formation	tuff	Stephanian (Doubinger 1970)
Saint-Etienne	France	? Assise des couches de St Etienne	shale	Stephanian (Becq Giraudon et al. 1995)
Knoviz	Czech Republic	? Hredle Member	shale	Stephanian (Opluštil et al. 2016)
Commentry	France	?	shale	Late Stephanian (Krings and Kerp 1999)
Sperbersbach	Germany	Goldlauter Formation	tuff	Asselian (Schneider and Romer 2010)
Kammerberg	Germany	Manebach Formation	grey facies of fluvial deposits	Asselian (Schneider and Romer 2010)
Oelsnitz	Germany	Hartensdorf Formation	?	Asselian (Schneider and Romer 2010)
Oberhof	Germany	Oberhof Formation	red and grey facies with up to 90 % of volcanics	Asselian/Sakmarian (Schneider and Romer 2010)
Winnweiler	Germany	Donnersberg Formation	tuff	Sakmarian/Artinskian (Schneider and Romer 2010)
Rotterode	Germany	Rotterode Formation	?	Sakmarian/Artinskian (Schneider and Romer 2010)

Thirty-eight specimens from the Museum d'Histoire Naturelle Jacques de la Comble in Autun, France, were borrowed by Naturalis Biodiversity Center in Leiden, the Netherlands, and detailed measurements of these fossils were carried out by use of a Zeiss SteREO Discovery.V20 microscope with a Zeiss AxioCam MRc 5 for photography, and the associated program AxioVision. The other collections were visited by the authors. Measuring was performed using the means available at each collection. Where digital measuring equipment was not available measurements were carried out by hand (i.e., with a binocular microscope and protractors). Photographs were made using a Lumix Panasonic DMC FZ 18 camera. Pictures of the fossils in the Prague collection were taken using the equipment available at the Paleontology department; measurements on these pictures were carried out in Leiden using AxioVision.

**Figure 1.**
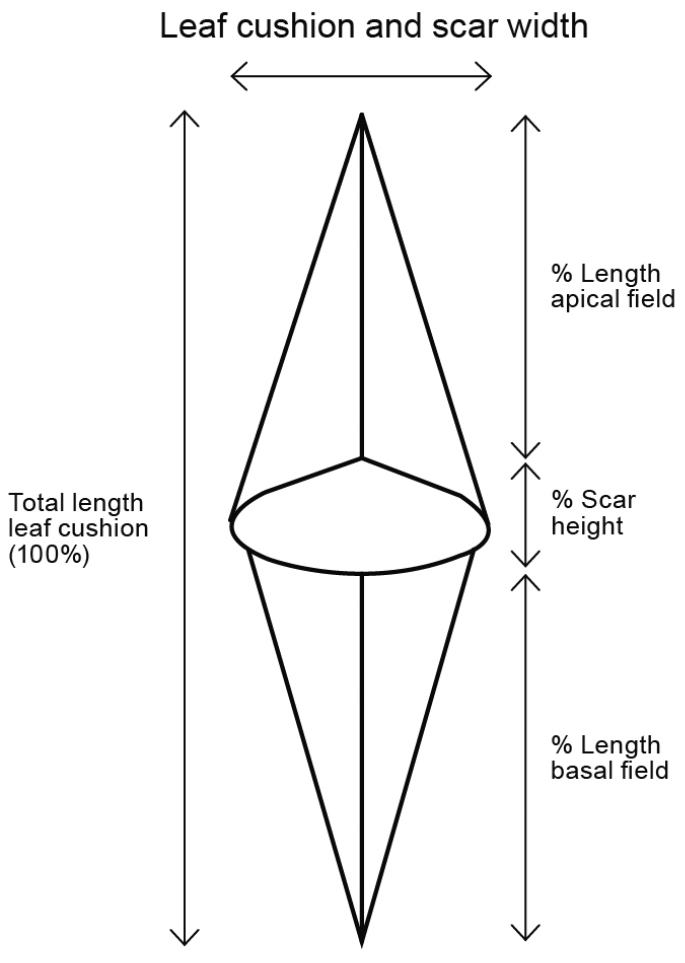
Schematic leaf cushion organisation: Total length and width leaf cushion, length apical and basal field leaf cushion, height and width leaf scar.

Measured characters for the stem (Suppl. material 2) were stem width, total, basal and apical leaf cushion length, leaf scar width and length (Fig. 1), and scar density. Measured characters of the leaf (Suppl. material 3) were the length of the middle leaf segment only (as basal and top parts were often missing), the width of each segment and its angle of bifurcation. Length and width of axillary shoots were measured (Suppl. material 4), as well as the length and width of seeds found in close proximity to the leaves, the size of their nucellus and the thickness of their integumentary coat (Suppl. material 5). The compilation of this primary data set was carried out using the paleontological statistics software package PAST 3.01 (Hammer et al. 2001).

## Results

Properties from the axis were recorded for 85 samples, properties from the leaves were recorded for 96 samples, and 19 axillary shoots and 15 seeds were measured.

### Stem

**Figure 2.**
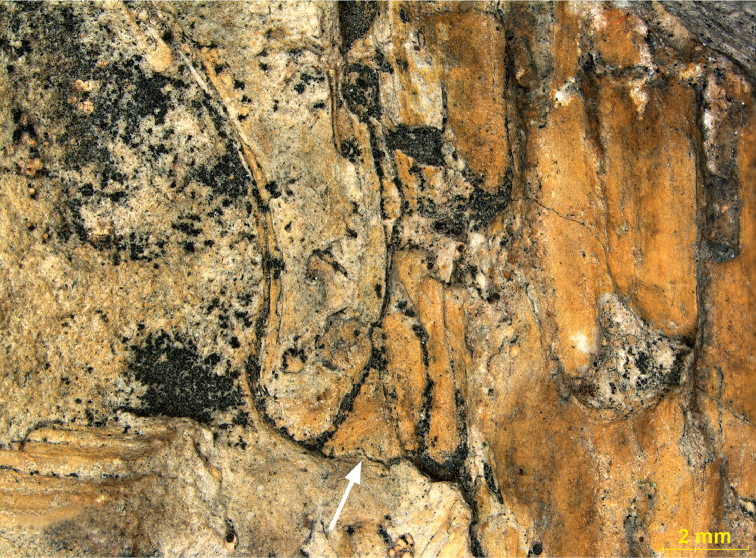
Side view of leaf attachment: sample 191, Mt Pelé. (Arrow indicates leaf attachment).

Stems are slender with a helical leaf arrangement. They are always fragmentary, but the longest fragment is 360 mm. The leaves are inserted at a perpendicular angle or even at a downward angle, after which the leaf departs at an acute angle thus commonly forming a pouch (Fig. 2).

The 85 measurements of the stem width indicate a diameter of 2 to 36 mm (mean 11 mm). Samples from the Mont Pelé are the smallest (on average 10 mm), while samples from Lubná, Oelsnitz and Ronchamp have on average wider stems (mean respectively 13, 16 and 17 mm).

### Leaf cushions

Leaf cushion length varies between 3 and 22 mm (mean 7 mm). Samples from Oelsnitz, Ronchamp and Lubná display longer leaf cushions (mean respectively 8, 9 and 12 mm). Leaf cushions are between 1.3 and 6.7 times longer than wide (mean 3.5, indicating an average width of 2 mm). The Oelsnitz samples have relatively broad leaf cushions (on average only 2.3 × longer than wide).

**Figure 3.**
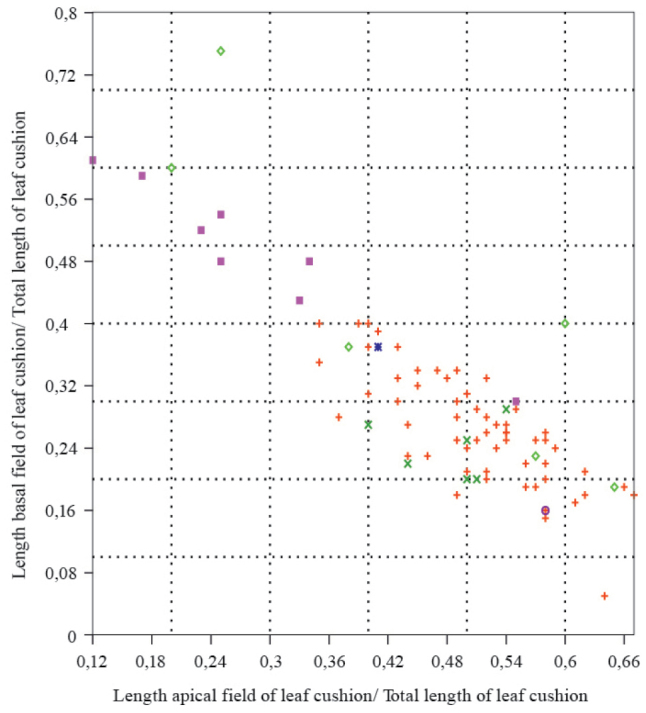
Diagram of the relative dominance of the basal over the apical leaf cushion field. Legend: Horizontal axis: Length apical leaf cushion field/ total length leaf cushion, vertical axis length basal leaf cushion field/ total length leaf cushion. Pink square: Lubná red cross: Mt Pelé, light green diamond: Ronchamp, dark green cross: Oelsnitz, dark blue star: St Etienne, purper circle: Kladno.

**Figure 4.**
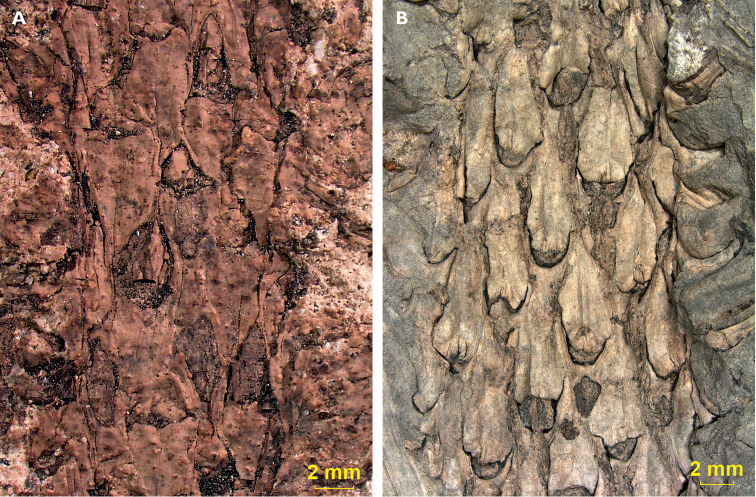
Variations in leaf cushion organisation: **A** Leaf cushion organisation in a specimen with a relatively dominant basal leaf cushion field, Sample E 06944, Lubná **B** Leaf cushion organisation in a specimen with relatively dominant apical leaf cushion field, Specimen 223, Mt Pelé.

The scar position in the present data determines the length of the basal and apical leaf cushion field (Fig. 1). On average the 20 % of the total leaf cushion length represented by the leaf scar is in the lower half of the leaf cushion, thus leaving 35% for the basal leaf cushion and 45% for the apical one. In plotting the size of the basal field relative to the total leaf cushion length against the relative size of the apical field to the total leaf cushion length (Fig. 3), it becomes apparent that the Lubná and two Ronchamp samples have a relatively large basal leaf cushion field (Fig. 4A), while the Mt Pelé, Oelsnitz and Kladno specimens have a small basal field and a relatively large apical field (Fig. 4B).

### Leaf scars

Leaf scars are broader than height. The leaf scar is apically rhombic acute, basally rhombic obtuse, with a height of 1-3 (mean 1.5) mm and a width of 1–3.5 (mean 2) mm.

**Figure 5.**
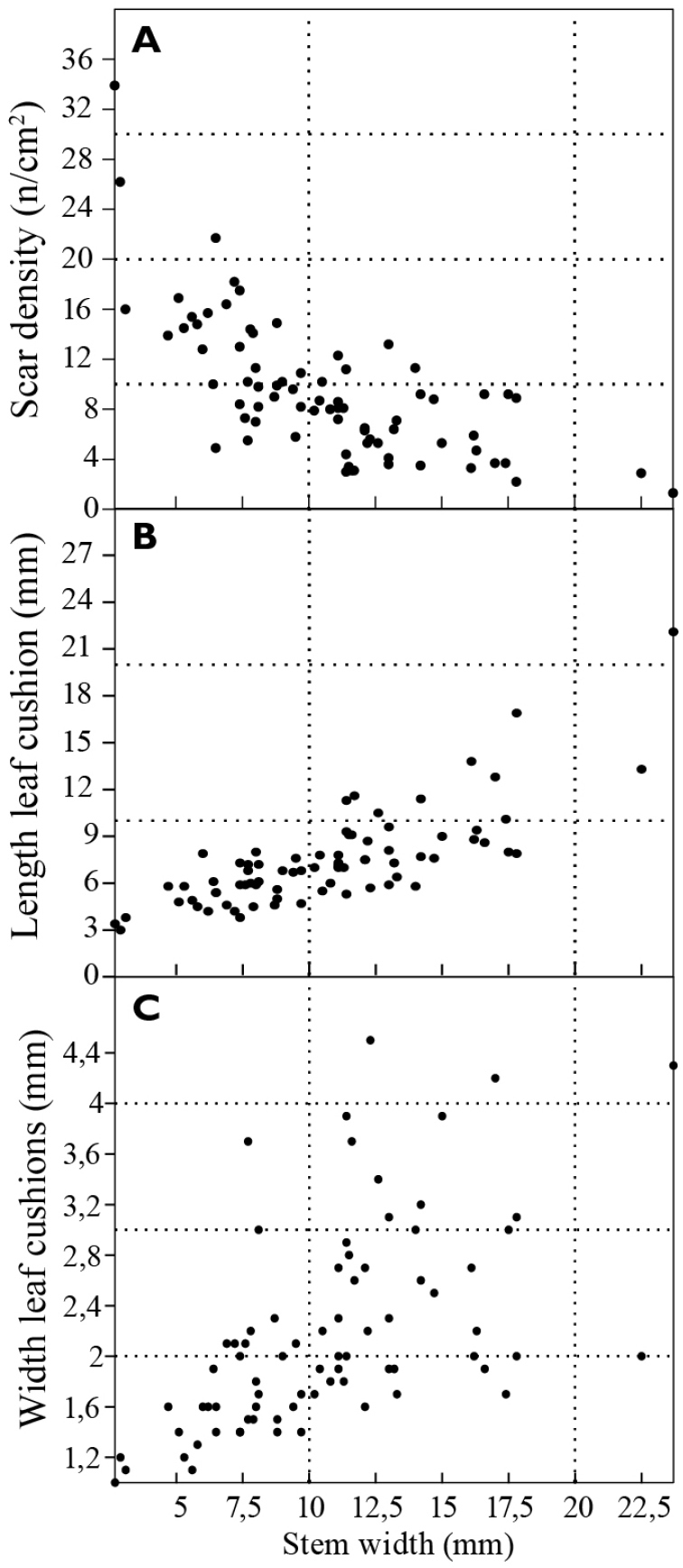
Changing properties with changing stem width: **A** Diagram of the scar density (in n/cm^2^) to the stem width (in mm) **B** Diagram of leaf cushion length (in mm) to stem width (in mm) **C** Diagram of leaf cushion width (in mm) to stem width (in mm).

The average leaf scar density varies strongly between 1 and 34/cm^2^ (mean 9/cm^2^). Scar density in the Mont Pelé material is highest (10/cm^2^), and is lower for the Lubná, Ronchamp and Oelsnitz samples (mean respectively 3, 3 and 7/cm ^2^). Furthermore, as can be inferred from the scar density plotted against stem width, these properties are correlated (Fig. 5A). The scar density decreases with the increase of the stem width. At the same time leaf cushion length and width increase with stem width (Fig. 5B,C).

### Leaves

**Figure 6.**
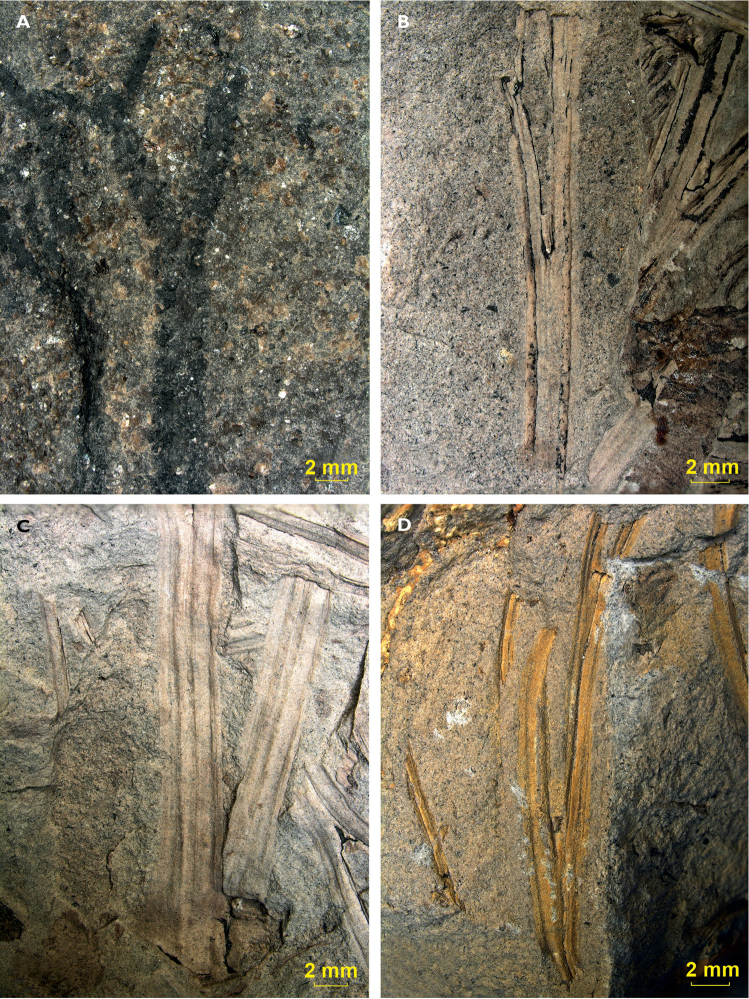
Leaf structures: **A** Leaf displaying two consecutive bifurcation angles, the second angle is larger than the first, sample 30, Ronchamp **B** Stomatal furrows as pronounced protrusions on the adaxial side of the leaf surface, note several thin striae between the furrows, sample 220 Mt Pelé **C** Stomatal furrows as pronounced depressions on the abaxial side of the leaf surface, note the midvein depression, sample 225 Mt Pelé **D** Venation in a *Dicranophyllum gallicum* leaf, note the trajectory of the midvein from the inner edge of the second leaf segment, gradually back to the centre position before the second bifurcation, sample 157, Mt Pelé.

The angle of bifurcation in leaf specimens ranged from 2 to 41 degrees for the first bifurcation, and 18 to 68 degrees for the second. The second angle is generally larger than the first (Fig. 6A).

As most leaves were incomplete, only the length of the middle segment could be measured. The length of the middle leaf segment varied between 1.4 and 87.0 mm (mean 18.2). The Mont Pelé samples had the smallest middle segment (mean of 8.4 mm), while Ronchamp, Lubná and Sperberbach second leaf fragments were larger (mean respectively 15.7, 19.7 and 19.9 mm).

The width of all three leaf fragments could be measured varying between 1.0 and 4.6 mm (mean 2.3 mm). While the width of the first non-bifurcated segment of Mt Pelé samples was relatively small (mean 1.9 mm), those of Ronchamp, Lubná and Sperberbach was relatively large (mean respectively 2.1, 2.9 and 2.9 mm). The width of the second segment varied between 0.6 and 2.5 mm (mean 1.4 mm) while, again, the width of the second segment of Mt Pelé samples was relatively small (mean 1 mm), and that of Ronchamp, Sperberbach and Lubná samples was relatively large (mean respectively 1.3, 1.8 and 2.0 mm). Finally the same was the case for the width of the third segment that varied between 0 and 2.1 mm (mean 0.5 mm). While the width of the third segment of the Mt Pelé samples was smallest (mean 0.2 mm), that of Ronchamp, Sperberbach and Lubná samples was relatively large (mean respectively 0.5, 0.9 and 1.4 mm).

### Leaf structure

Two rather distinct cuticular patterns on *D. gallicum* leaves can be observed on the different sides of the leaf. In a few specimens, two furrows were very clearly pronounced as protrusions on the impressed surface and no midvein was apparent (Fig. 6B); instead, we observed several thin striae between the furrows. As the furrows are documented to be located on the abaxial side of the leaf (Barthel 1977, Meyen and Smoller 1986), these leaves are identified as impressions of the abaxial side of the leaf. Impressions of the adaxial side of the leaf form the second leaf pattern that consists of a narrow midvein intrusion and two wide depressions which indicate the location of the underlying furrows pushing the adaxial leaf surface upwards (Fig. 6C). The furrows are between 0.3 and 0.4 mm (mean 0.35 mm). After its first bifurcation the midvein was observed to follow a distinct trajectory into the second leaf segment (Fig. 6D). It splits some distance before the actual bifurcation of the leaf and then follows the inner edge of the second segment while gradually returning to the centre position in the leaf sheet. Simultaneously, a new furrow appears alongside it.

**Figure 7.**
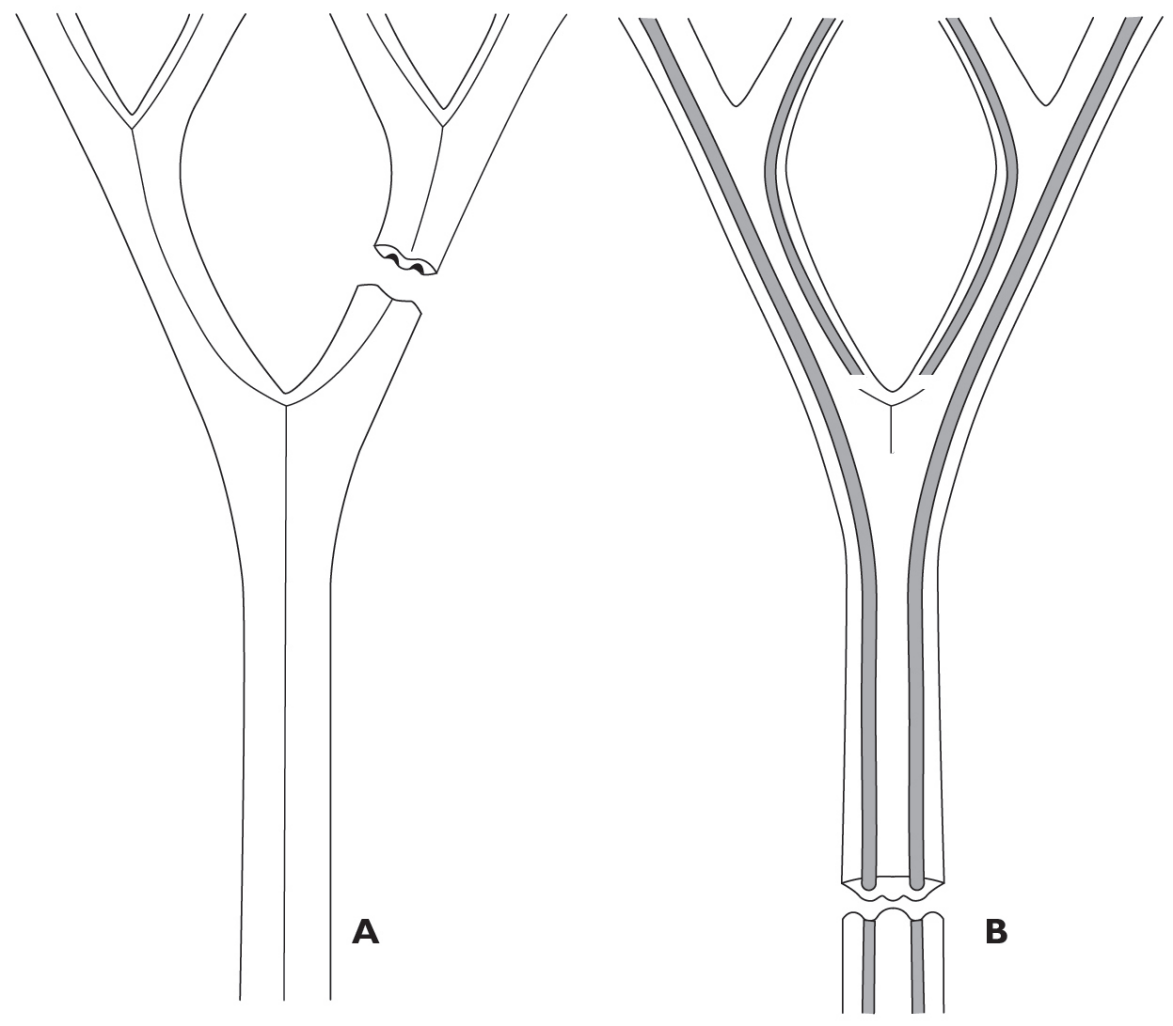
Diagram of leaf structure: **A** Reconstruction of the adaxial leaf side of *Dicranophyllum gallicum*, note the path of the midvein in the second segment of the leaf **B** Reconstruction of the abaxial leaf side of *Dicranophyllum gallicum*, note the prominent furrows and the absence of a clear midvein.

Based on present observations of the adaxial and abaxial sides of the leaves of *D. gallicum* (Fig. 6B, C), it was possible to reconstruct the cross section of the leaf (Fig. 7A, B). As the furrows are visible on both sides of the leaf, the leaf of *D. gallicum* is expected to have been rather thin. The furrows on the underside of the leaf push the upper leaf surface upwards, causing the depressions in impressions of the adaxial leaf surface.

**Figure 8.**
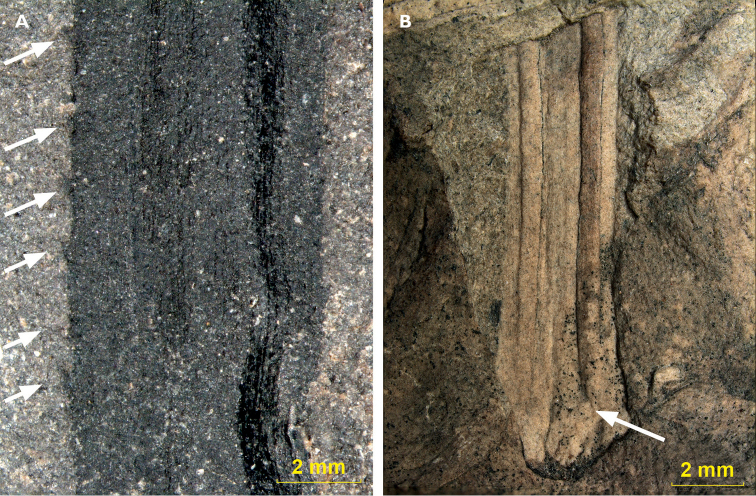
Leaf details: A Marginal microdenticulation, sample 34, Ronchamp (arrows indicate microdenticulation). B. Basal part of the leaf showing that the two stomatal furrows do not run all the way down in the leaf base, sample 1529, Mt Pelé (arrow indicates origin of furrow).

On some compression fossils marginal microdenticulation is preserved (Fig. 8A).

### Attachment of the leaves to the stem

Reconstructing the attachment of leaves to the stem is difficult, as these two organs are commonly found separated from each other. Several specimens of *D. gallicum* do show a stem fragment with remnants of attached leaves, in which the leaf follows a specific curve as it escapes from the stem. There is little information on how this trajectory appears on leaf remains, as most leaves with clear venation are broken off and left their basal portion behind. An exception was found on specimen 1529 of Mt Pelé (Fig. 8B): this is an impression of the abaxial side of a leaf. The basal part of the leaf shows that the two furrows do not run all the way down in the leaf base, but find their origin more centrally to the leaf scar. Extrapolation of the orientation of the two furrows relative to the midvein indicates an origin for the two furrows near the basal portion of the leaf scar.

### Axillary shoots

**Figure 9.**
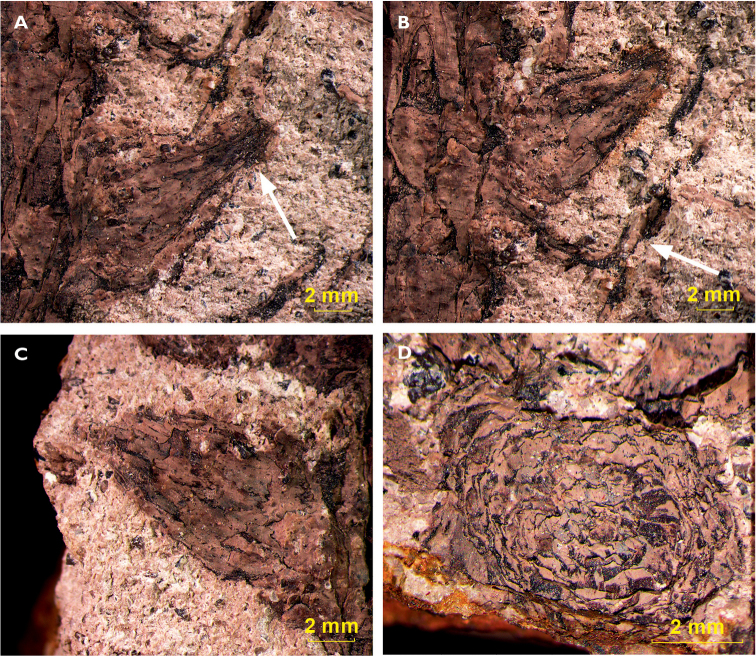
Axillary shoots: **A** Axillary shoot with apical widening (indicated by arrow), E 06946, Lubná **B** Axillary shoot with subtending leaf (indicated by arrow), sample E 06946, Lubná **C** Axillary shoot without broad apical widening, sample E 06950, Lubná **D** Cross section of axillary shoot, sample E 06946, Lubná.

Well-preserved axillary shoots were found in the collections of the Museum of Natural History in Prague. Various shoots are attached to the stem of *Dicranophyllum gallicum* specimens from the Lubná locality (Fig. 9A-D). They are composed of elongated scales of 6 mm in length and 2 mm in width and are commonly positioned in the leaf axil. Although they have a general conical shape, their apical end is often widened (Fig. 9A). Their length varies between 4.0 and 14.0 mm (mean 8.0 mm), and their width varies between 2.5 and 7.0 mm (mean 4.0 mm) (Suppl. material 4). A cross section of one of these structures (Fig. 9D) could also be observed. It clearly displays the helical arrangement of the scales forming it. Some specimens from the Mont-Pelé locality also show axillary shoots.

### Seeds

**Figure 10.**
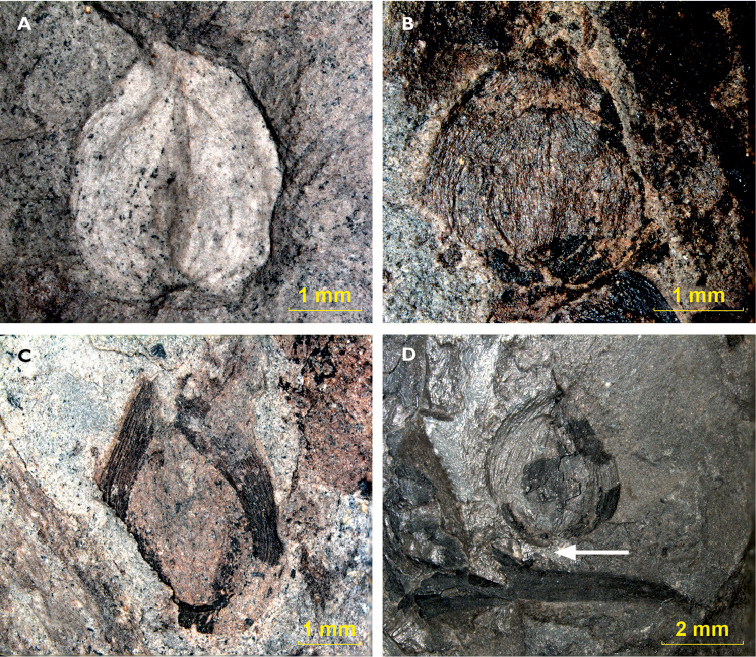
Seeds: **A** Seed with notched chalaza, sample 1529, Mt Pelé **B** Striate seed with visible pollen chamber, sample 75, Mt Pelé **C** Seed with visible micropyle, sample 75, Mt Pelé **D** Seed possibly on first leaf segment, sample 1362, Ronchamp (arrow indicates interrupted organic bridge).

A total of 15 seeds was observed on the specimens in our study (Supplementary File 5). They were found dispersed on specimens from the Mont-Pelé locality, with one exception from the Ronchamp locality (specimen 1362). Seeds have a conical to ovate shape, 2.6-3.8 mm long (mean 3.4 mm) and 2.8-3.8 mm wide (mean 3.1 mm), usually longer than wide. A nucellus can often be observed and is 1.0-1.5 mm long (mean 1.2 mm) and 0.7-1.2 mm wide (mean 1.0 mm). The seed base is rounded, the chalaza part is flattened to notched or cordate (Fig. 10A). The seed surface is striate (Fig. 10B). The apex is notched with what appears to be a (sometimes wide) micropyle (Fig. 10C). In a single seed (Fig. 10B) a triangular cavity which could be interpreted as a pollen chamber is seen in the seed. The integumentary coat is thick (200 µm), apically slightly thicker than basally. Two seeds are positioned next to a *Dicranophyllum gallicum* leaf, one of which shows an organic bridge between the leaf and the seed, but the bridge is interrupted (see Fig. 10D).

## Discussion

### Stem

The stem diameter recorded in the literature hovers around the mean values of 11 mm recorded here such as 9.0 mm for D. gallicum var. parchemineyi (Renault and Zeiller 1888), 10 mm for the German *D. gallicum* described by Barthel (1977) and 8 to 14 mm for the 7 samples from La Magdalena (Castro Martínez, 2005). Samples from Lubná, Oelsnitz and Ronchamp are wider, sample 10472 from Ronchamp being 36 mm wide. This indicates that the variability in the present European stem selection is much higher than what was recorded until now.

### Leaf persistence and growth patterns

Leaves were found attached to stems of various sizes, implying that the plant had leaves covering most of the stem. Nonetheless, stem 221 in our study material shows a transition from large, well-developed leaf cushions to small, poorly defined leaf cushions. This transition gives an indication that the more apical part of these stems was still developing, while the more basal part was mature. Periodic growth patterns have not been observed on any stems, not even on the longest stem fragments of *D. gallicum*. This suggests that stem growth in *D. gallicum* never halted, that is, it was more or less continuous.

Many stem fragments are gently curved. Although we have not analysed this character in detail, it makes sense to expect that thicker stems are more resistant to bending, while slender stems are more flexible (also being younger). The majority of stems in the *Dicranophyllum gallicum* material is slender and curved to some extent, indicating that during its life the plant was probably flexible.

Grand’Eury (1877), Renault and Zeiller (1888) and Barthel (1977) considered the leaves to be persistent. The density of attached leaves appears to have been the largest in the apical regions, where establishing the details of the leaf morphology was often difficult as the leaves were preserved on top of each other. In the present material specimens with thick stems still had leaves attached (e.g. specimen 223), while other stems were completely devoid of leaves, suggesting leaf persistence related to an ecological factor.

### Leaf cushion

Leaf cushion length and width from literature, respectively 4-7 mm and 2-3 mm (Zeiller 1880, Renault and Zeiller 1888, Castro Martínez 2005), is shorter than the mean values found in the data set described here (respectively 7 to 2 mm). However, it has been said to vary considerably (Renault and Zeiller 1888, Barthel 1977).

The dominant leaf cushion organisation with a large apical field is in contrast with the leaf cushion organisation given in Grand’Eury (1877). He described the leaf scar as positioned in the upper third of the leaf cushion, thus resulting in a very small apical field. Renault and Zeiller (1888) even placed the leaf scar in the upper quarter of the leaf cushion. Scrutiny of the illustrations of Grand’Eury (1877, plate XIV, fig 10, in particular) confirms a position of the leaf scar in the upper half of the leaf cushion, yet in Zeiller (1880, plate CLXXVI, fig 1) it cannot be established. In Renault and Zeiller (1888, plate LXX, fig 8 and plate LXXI, fig. 5), on the contrary, the scar is illustrated as either positioned basally or centrally in a similar way as for the bulk of the collections described herein.

As only the Lubná and the two Ronchamp samples have a well-developed basal leaf cushion field, these specimens are best comparable to the figured paratype of Grand’Eury (1877, plate XIV, fig 10). This is in contrast with the samples from Mt Pelé where, inversely, the apical field dominates over the basal field.

### Leaf bifurcation angle

Renault and Zeiller (1888) described the angle of leaf bifurcation as 30° for the first bifurcation and 40° for the second. Barthel (1977) reported angles between 6° and 30°. The variation in angle size for the material described herein is much wider (2° to 68°) than what is given in the literature, yet the first angle is, indeed, commonly smaller than the second.

### Leaf sizes

Earlier *Dicranophyllum gallicum* descriptions indicate that the total leaf length varied between 33 and 60 mm, with on average 15-20 mm for the first segment, 10-15 mm for the second and 8 to 10 mm for the third (Zeiller 1880, Renault and Zeiller 1888, Barthel 1977, Castro Martínez 2005). Doubinger et al. (1995) considered that the leaves can reach a length of 200 mm. The present material, based on our measurements of the second segment (mean 18.2 mm), was slightly larger than what was described earlier. Lubná, Sperberbach, Manebach and Ronchamp leaves were even larger (on average respectively 20, 17, 20 and 16 mm), while the Mt Pelé samples were, again, relatively small (8 mm).

The leaf width from earlier studies varied between 1.5 and 2.0 mm (Renault and Zeiller 1888) or between 2.0 and 3.0 mm (Barthel 1977). In the present material the leaf width varies between the same values.

### Leaf structures

*Dicranophyllum gallicum* found in the Erzgebirge has small abaxial stomatal furrows of 0.2 to 0.25 mm wide. The furrow width in the present material was slightly larger (0.35 mm). Thin striae as found here between the two furrows have also been described earlier as five strong lineations in *D. gallicum* samples from the Goldlauterer Schichten (Barthel 1977).

The trajectory of the midvein presented above (Figs 6, 7) is one where the midvein approaches the leaf margin at the leaf bifurcation, to later diverge from the leaf margin and to again reach the central part of the leaf. This midvein pattern was not described earlier and is depicted differently in Barthel (1977) as splitting a few millimetres from the leaf margin in such a way that the two new veins always remain central to the leaf.

### Leaves development

Barthel (1977) describes a process of leaf development as starting with simple unbifurcated leaves, after which the leaves produce a first, terminal bifurcation and, finally, a second bifurcation. According to De Lima (1888) a leaf can bifurcate three times.

**Figure 11.**
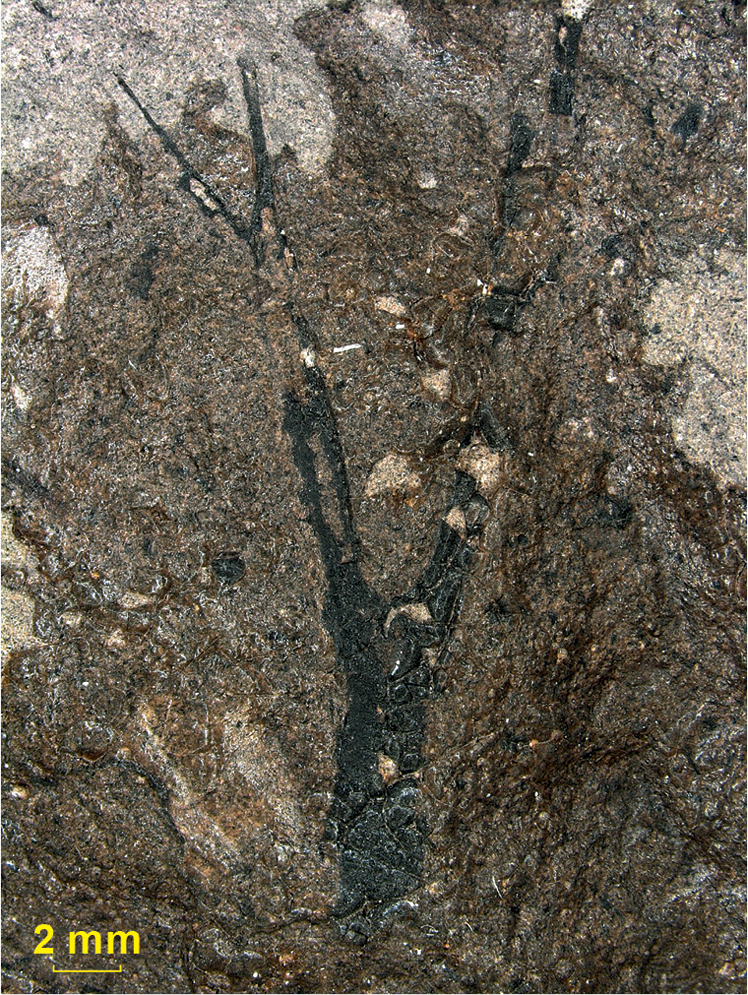
Possible juvenile leaf, sample 28, Mt Pelé.

Based on present material it is difficult to conceive such a leaf development, as no clear simple leaves on any *D. gallicum* specimen was observed. Incomplete leaves without bifurcations were observed, these could be associated to *D. gallicum* based on venation properties, that is, the clear presence of furrows, but it was not possible to make statements about whether those leaves bifurcate or are, indeed, simple leaves. A few leaves do display a size and shape that suggests an early stage of development (Fig. 11), but these already have two bifurcations. Ultimately, our material provides too little evidence to make an assumption about how the leaves developed.

### Axillary shoots

Renault and Zeiller (1888), in describing a sample from Puits Forêt, indicated a charcoalified body formed by scales of 5 to 6 mm long and 2 to 3 mm wide attached to the stem. These scales are comparable in size to the scales described from the axillary shoots described above. Renault and Zeiller (1888) described the charcoalified body as seemingly forming a bud that had contained microsporangia, while the whole shoot was reminiscent in shape and size to cordaite male organs.

*Dicranophyllum hallei* also displays axillary shoots and Barthel and Noll (1999) suggested that these represent fertile cones (either male or female) while Noll (2011) found a dispersed female cone of *D. hallei*. In *Barthelia furcata* axillary shoots subtended by a forked bract represent ovuliferous dwarf shoots (Rothwell et al. 2005). In Anderson et al. (2007, fig. 2, p. 114), axillary shoots of *Dicranophyllum gallicum* are described as male cones, but in Meyen (1987) the axillary shoots, compared to axillary buds in *Mostotchkia*, are said to have been misinterpreted as microstrobili. From the present material and from what was described earlier, no unequivocal proof of the true nature of these axillary structures can be deducted.

### Seeds

Seeds were already mentioned by Grand’Eury (1877) as very small, conical shapes positioned just above rather than at the leaf axil. Seeds have been found attached to the first leaf segment for Dicranophyllum gallicum var. parchemineyi, and described to be 4 mm long and 3 mm wide (Renault and Zeiller 1888). These sizes differ slightly from what is observed in present material (on average 3.4 long and 3.1 wide), but certainly are in the same order of magnitude. The nucellus described in the results above is smaller (mean 1.2 long and 1.0 mm wide) than what is described in Renault and Zeiller (1888) that is, 2.8 to 2.0 mm. The integumental coat, while being apically thicker in a similar way to what was described earlier, does not reach the 1 mm indicated in Renault and Zeiller (1888). These differences in size can be hypothesized to relate to the Mt Pelé material being generally smaller than was described earlier. On the other hand, because seeds are propagules and conical seeds with a nucellus and a pollen chamber are produced by numerous divergent taxa, the occurrence of these seeds in both the Ronchamp and the Mt Pelé samples and their resemblance to the seeds described by Grand’Eury (1877) and Renault and Zeiller (1888) have to be considered here as probably coincidental and further advances on *Dicranophyllum gallicum* will have to await new and better finds.

Obviously, finding and describing the original material of *Dicranophyllum gallicum* Renault & Zeiller, 1888, would give unequivocal proof of the nature and organisation of the female fertile structures, but, in spite of a specific search for it, this sample cannot be located. Here we only have the interrupted organic bridge between the leaf base and the seed (Fig. 10D). As D. gallicum var. parchemineyi was illustrated with its seeds attached to the first, non-bifurcated segment of the leaf (Renault and Zeiller 1888, plate LXXI, fig. 5), it is tempting to consider this seed to be attached to the leaf as well, given its proximity and orientation towards the leaf. However, it is not possible to find stronger evidence for any further physical connection between the leaf and seed.

### Ontogenetic variability

As seen in the results, the Mt Pelé samples are relatively small in leaf and stem size, have a normal leaf cushion length, have the highest leaf scar density and have a dominance of the apical over the basal leaf cushion field. The Lubná, Ronchamp and Oelsnitz samples, in contrast, relatively have a large stem and a short leaf cushion with a large basal leaf field and low scar density.

Such differences in features could indicate a phylogenetic differentiation, where the large Lubná samples are ancestral to smaller *Dicranophyllum gallicum* specimens, Lubná is amongst the oldest (from the Westphalian C, Table 1) localities in the present series, yet the *D. gallicum* from Kladno originates from the same Radnice Member as the Lubná samples do, yet ranges with its smaller dimensions and leaf cushion organisation with the rest of the French and German *D. gallicum*. It was demonstrated that scar density was negatively correlated to stem width (Fig. 5A), while leaf cushion length and width correlates positively to stem width (Fig. 5B, C). Lubná samples, have relatively large stems, low scar density and long and wide leaf cushions and are considered to have represented matured plants, having had the time to grow thicker stems, and longer and wider leaf cushions, thus resulting in a reduced scar density. The relative dominance of the basal over the apical leaf cushion field (Fig. 3), typifying the Luba specimens, consequently is hypothesized here to relate to an ontogenetic development. The paratype (Grand’Eury 1877, plate VIV, fig. 10), the samples from Lubná and two samples from Ronchamp are considered to have been more mature specimen, while the Mt Pelé samples, with thin stems, relatively elongated leaf cushions and a high leaf scar density, are considered juvenile forms. As there is no systematic relation between lithology (Table 1), considered here as a reflection of the environmental setting, and specimen dimensions, ecology is not considered to play a part in the variability described herein.

### Reconstruction

For the reconstruction of the habit of *Dicranophyllum gallicum* using Niklas' (1994) method, only the diameter of the plant is required. By using this method it has to be assumed that *D. gallicum* was a self-supporting plant, as the method was shown to be inapplicable to liana species (Niklas 1994, p. 1237). Indeed, *D. gallicum* shows no features like climbing hooks (see, for example, Krings and Kerp 1999 or Krings et al. 2003a).

In spite of Grand’Eury’s (1877) early suggestion that a woody body formed underneath the leaf cushions and Barthel's (1977) description of *Dicranophyllum gallicum* as a woody plant, there is some uncertainty as to the true woodiness of the stem. Assuming that *D. gallicum* was, indeed, a woody plant and taking the largest stem diameters of 36 mm, it is calculated to have reached a height of at least 4.5 m (Table 2).

**Table 2.** Reconstruction of stem height.

Maximum width (m)	Result (m)	Wood type	Niklas' formulas
	4.49	woody	10^(1.59+0.39 (Log 10) (Stem width))-0.18 (Log 10) (Stem width)^2)
0.036	3.37	nonwoody	10^(2.51+1.41 (Log 10) (Stem width))-0.03 (Log 10) (Stem width)^2)
	3.37	intermediate	10^(1.81+0.7 (Log 10) (Stem width))-0.13 (Log 10) (Stem width)^2)
**Average width (m)**			
	1.38	woody	10^(1.59+0.39 (Log 10) (Stem width))-0.18 (Log 10) (Stem width)^2)
0.0111	0.73	nonwoody	10^(2.51+1.41 (Log 10) (Stem width))-0.03 (Log 10) (Stem width)^2)
	0.88	intermediate	10^(1.81+0.7 (Log 10) (Stem width))-0.13 (Log 10) (Stem width)^2)

**Figure 12.**
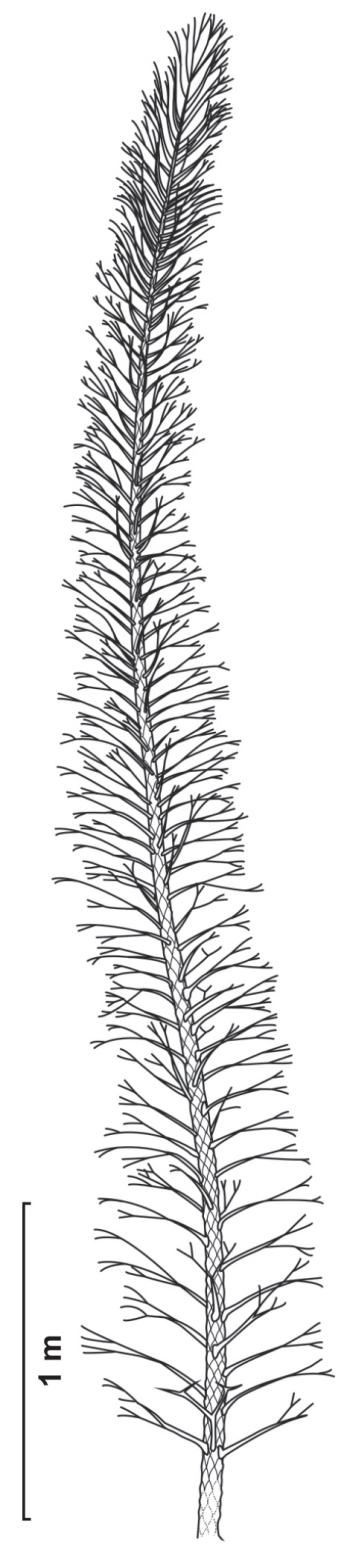
Reconstruction of *Dicranophyllum gallicum*.

This expected height could increase if *D. gallicum* were shown to have branched. Grand’Eury showed a single specimen with a branching stem fragment (Grand’Eury 1877, plate XIV, fig. 8), and reconstructed the species accordingly as a small tree (1877, tableau de vegetation D, bottom left). However, no other *D. gallicum* fossils showing branching have been found since and none of the specimens in our study showed branching; Grand’Eury (1877) figured specimen with a bifurcating branch is the only one showing this feature. This indicates that branching is either a rare occurrence in *D. gallicum* or the plant did not branch at all, thus suggesting that the specimen from Grand’Eury (1877) would be of a different species. For our reconstruction of *D. gallicum*, we propose that the plant had a simple, unbranched stem, which reached 4.5 meters in height (Fig. 12). This is comparable to the reconstruction of *Dicranophyllum hallei* by Barthel et al. (1998), in which a nearly complete, unbranched shoot of 2 m long is described. The stem widths of *D. hallei* specimens we observed were usually slightly greater than the *D. gallicum* measurements, indicating that *D. hallei* grew taller than *D. gallicum*.

### Taxonomic affinity

Finding similarities between the pollination and fertilization process, and the presence of the retained motile sperms in *Ginkgo* and the Cycadales, Thomas and Spicer (1987) suggested the existence of a group of primitive pteridosperms for the common ancestor of the later. Such a group of primitive pteridosperms holding *Dicranophyllum gallicum* supports the views of Archangelsky and Cúneo (1990) of the representing an order of primitive gymnosperms. Rothwell et al. (2005) in their study of *Hanskerpia* found *Dicranophyllum hallei* to be positioned between the Cordaitales and the at the base of the early conifers. Given that Renault and Zeiller (1888) considered *Dicranophyllum gallicum* to possibly have had male axillary shoots reminiscent of the microstrobili in Cordaitales, such a position for a member of the at the very base of the early conifers would be corroborated by an interpretation of the present axillary shoots as pollen cones, but in the absence of any unequivoval proof of them representing pollen cones, the axillary shoots are considered here as vegetative buds. As such this interpretation does not differ from the description given in Rothwell and Mapes (2001). They described *D. gallicum* as being represented by female fertile structures only and they considered it challenging to give a systematic assignment to early coniferophytes in general, e.g. the *sensu* Meyen (1987).

### Environmental context

**Marginal microdenticulation**

Marginal microdenticulation (Fig. 8B) may indicate adaptation to a xeric environment, in which the teeth aid the plant in maintaining its internal temperature or absorb nutrients, as found in some extant xerophilic species (e.g. Benzing 1976, Benzing et al. 1976). However, marginal teeth are known to serve different functions for plants such as defence against herbivory (Krings et al. 2003b) and storage of minerals (Broadhurst et al. 2004).

**Stomatal furrow**

Although it was not possible with the present material to perform a cuticular analysis on the leaves of *Dicranophyllum gallicum*, Barthels' (1977) work shows that the stomata of this species occur in the embedded abaxial furrows. Jordan et al. (2008) showed that encryption of stomata in grooves (as found in *Dicranophyllum*) has evolved separately several times in a number of species of Proteaceae that are known from arid to semi-arid environments. Archangelsky et al. (1995) associated the hypostomatic leaves of the Cretaceous cycad *Pseudoctenis ornata* Archangelsky to the volcanic environment in which it occurred. The hypostomatic nature of the leaves provides no certainty about the vegetation density, which may be open as well as closed (Jordan et al. 2014). Moreover, Parkhurst (1978) explained that stomatal distribution is not primarily caused by environmental factors, but can be seen as an indicator of leaf thickness (thick leaves generally being amphistomatous).

**Other plant fossils found co-occurring on the *Dicranophyllum gallicum* samples**

Information about associated plant species occurring beside *Dicranophyllum gallicum* mainly comes from the tuffaceous material of the Mont-Pelé locality. The specimens from this locality had fragments of *Calamites*, *Pecopteris*, *Nemejcopteris*, *Alethopteris*, *Neuropteris*, *Sphenopteris*, *Cordaites*, and the seeds *Pachytesta* and *Samaropsis* associated with them. Also, on specimen E 06948 from the Lubná locality, a megasporangium of an *Omphalophloios* sp. was found. These genera are predominately known from the late Pennsylvanian tropical forests of Euramerica and, as *Dicranophyllum gallicum* was maximally 4.5 m high, it is suggested here that the plant stood in the shade of its taller associates. *Omphalophloios feistmantelii* is also 2-3 m in height and has been interpreted as a plant able to rapidly colonize local habitats, preferring peat and mixed peat-clastic swamps (Bek et al. 2015). *Dicranophyllum gallicum*, because of its comparable size and co-occurrence, is suggested here to have occupied the same or similar habitat.

**Sediment**

*Dicranophyllum gallicum* is as often preserved in tuffs as in shales (Table 1). Assuming the specimens preserved in tuffs are (par-) autochtonous, *D. gallicum* may have thrived in the mesic-xeric areas from a volcanic slope with active volcanism. The specimens found in shales are considered to have grown in wetter areas, as suggested in Anderson et al. (2007) for , in riparian or lake-side environments. Yet the occurrence in tuffs supports a more ruderal behaviour that the association with an *Omphalophloios* sp. already suggests, thus indicating an opportunistic colonization strategy. According to Wagner (2005) the paucity of *Dicranophyllum* finds may have indicated that it had ecological requirements differed from those prevailing in the 'coal measures' and rather belonged to extra basinal, well-drained soils. Considering *D. gallicum* is found both in shales and in tuff, an opportunistic colonization strategy as for *Omphalophloios* sp. is preferred here.

## Conclusions

It appears that the variability of *Dicranophyllum gallicum* in stem size, leaf cushion organisation, leaf size and bifurcation angle is much wider than what was presented in earlier studies. The variability in leaf cushion organisation with either the dominance of the apical or the basal leaf cushion field is newly described herein. Dominant apical leaf cushion fields are generally found on specimens with small stems, relatively long leaf cushions and a relatively high leaf scar density, while well-developed basal leaf cushion fields commonly occur on specimens with a broad stem, relatively wide leaf cushions and relatively low scar density. As scar density and stem width are gradually and negatively correlated, while the two stem types have the same stratigraphic occurrence, but divergent lithologies, the variability they represent is considered to point towards ontogenetic rather than phylogenetic or ecologic variability. The smaller stems with higher scar density and elongated leaf scar with a dominant apical field are considered juvenile while the larger stems with lower scar density, relatively broad leaf scars with well-developed basal field are considered mature specimens. The newly described Lubná samples chiefly represent mature specimens.

## Supplementary materials

Supplementary material 1 List of samples holding the *Dicranophyllum gallicum* specimen considered in present analysis. Data type: Microsoft Excel Worksheet (.xlsx) Explanation note: List of samples holding the *Dicranophyllum gallicum* specimen considered in present analysis. The samples are listed by collection, specimen number and locality. This dataset is made available under the Open Database License
(http://opendatacommons.org/licenses/odbl/1.0/). The Open Database License (ODbL) is a
license agreement intended to allow users to freely share, modify, and use this
Dataset while maintaining this same freedom for others, provided that the original
source and author(s) are credited.Jorik Van Der Pas, Linda Poppe, Isabel M. Van Waveren

Supplementary material 2 Measurements on *Dicranophyllum gallicum* stem. Data type: Microsoft Excel Worksheet (.xlsx) Explanation note: Measurements on *Dicranophyllum gallicum* stem: stem width, leaf scar length and width, leaf cushion length and width and apical and basal leaf cushion length in mm and leaf scar density in number per cm^2^. This dataset is made available under the Open Database License
(http://opendatacommons.org/licenses/odbl/1.0/). The Open Database License (ODbL) is a
license agreement intended to allow users to freely share, modify, and use this
Dataset while maintaining this same freedom for others, provided that the original
source and author(s) are credited.Jorik Van Der Pas, Linda Poppe, Isabel M. Van Waveren

Supplementary material 3 Measurements of *Dicranophyllum gallicum* leaves. Data type: Microsoft Excel Worksheet (.xlsx) Explanation note: Measurements of *Dicranophyllum gallicum* leaves: length second segment, width first, second and third segment in mm. This dataset is made available under the Open Database License
(http://opendatacommons.org/licenses/odbl/1.0/). The Open Database License (ODbL) is a
license agreement intended to allow users to freely share, modify, and use this
Dataset while maintaining this same freedom for others, provided that the original
source and author(s) are credited.Jorik Van Der Pas, Linda Poppe, Isabel M. Van Waveren

Supplementary material 4 Measurements of *Dicranophyllum gallicum* axillary shoots. Data type: Microsoft Excel Worksheet (.xlsx) Explanation note: Measurements of *Dicranophyllum gallicum* axillary shoots: maximal length, maximal width, apex width in mm. This dataset is made available under the Open Database License
(http://opendatacommons.org/licenses/odbl/1.0/). The Open Database License (ODbL) is a
license agreement intended to allow users to freely share, modify, and use this
Dataset while maintaining this same freedom for others, provided that the original
source and author(s) are credited.Jorik Van Der Pas, Linda Poppe, Isabel M. Van Waveren

Supplementary material 5 Measurements of seeds co-occurring with *Dicranophyllum gallicum*. Data type: Microsoft Excel Worksheet (.xlsx) Explanation note: Measurements of co-occurring seeds: seeds length and width and nucellus length and width in mm. This dataset is made available under the Open Database License
(http://opendatacommons.org/licenses/odbl/1.0/). The Open Database License (ODbL) is a
license agreement intended to allow users to freely share, modify, and use this
Dataset while maintaining this same freedom for others, provided that the original
source and author(s) are credited.Jorik Van Der Pas, Linda Poppe, Isabel M. Van Waveren
